# Graphene analogue in (111)-oriented BaBiO_3_ bilayer heterostructures for topological electronics

**DOI:** 10.1038/s41598-017-19090-3

**Published:** 2018-01-11

**Authors:** Rokyeon Kim, Jaejun Yu, Hosub Jin

**Affiliations:** 1 Center for Correlated Electron Systems, Institute for Basic Science (IBS), Seoul, 08826 Republic of Korea; 2 Department Physics and Astronomy, Seoul National University, Seoul, 08826 Republic of Korea; 3 Department of Physics, Ulsan National Institute of Science and Technology (UNIST), Ulsan, 44919 Republic of Korea

## Abstract

Topological electronics is a new field that uses topological charges as current-carrying degrees of freedom. For topological electronics applications, systems should host topologically distinct phases to control the topological domain boundary through which the topological charges can flow. Due to their multiple Dirac cones and the π-Berry phase of each Dirac cone, graphene-like electronic structures constitute an ideal platform for topological electronics; graphene can provide various topological phases when incorporated with large spin-orbit coupling and mass-gap tunability via symmetry-breaking. Here, we propose that a (111)-oriented BaBiO_3_ bilayer (BBL) sandwiched between large-gap perovskite oxides is a promising candidate for topological electronics by realizing a gap-tunable, and consequently a topology-tunable, graphene analogue. Depending on how neighboring perovskite spacers are chosen, the inversion symmetry of the BBL heterostructure can be either conserved or broken, leading to the quantum spin Hall (QSH) and quantum valley Hall (QVH) phases, respectively. BBL sandwiched by ferroelectric compounds enables switching of the QSH and QVH phases and generates the topological domain boundary. Given the abundant order parameters of the sandwiching oxides, the BBL can serve as versatile topological building blocks in oxide heterostructures.

## Introduction

The emergence of physical degrees of freedom and their easy manipulation has led to corresponding fields of electronics, such as spintronics^1^, valleytronics^2^, and plasmonics^3^. As a unique degree of freedom appearing only at the domain boundary between two different topological phases, a topological charge^4^ can give rise to *topological electronics*. For topological electronics, whereby information is carried by the topological charge through the topological domain boundary^5,6^, at least two distinct topological phases should be accessible and controllable within a system. In this regard, a gap-tunable graphene-like electronic structure^7,8^ is an ideal candidate for topological electronics applications. It has been suggested that various topological phases can be hosted by opening the band-gap of a Dirac cone via spin-orbit coupling (SOC) or symmetry-breaking; the quantum spin Hall (QSH) phase can be induced by SOC^9^, the quantum valley Hall (QVH) phase by breaking the sublattice symmetry^10^, and the quantum anomalous Hall phase by breaking the time-reversal symmetry^11^.

**Figure 1:**
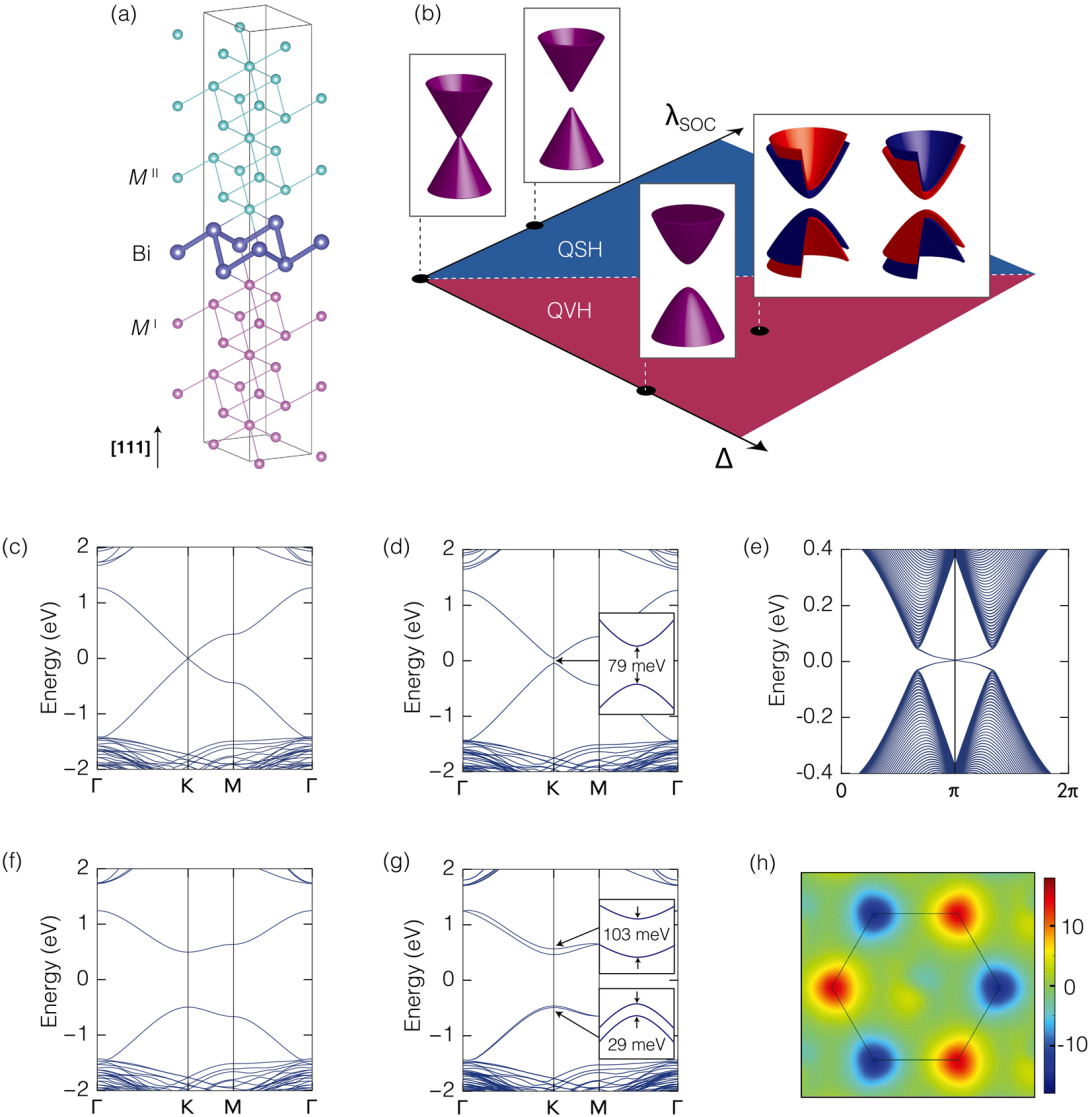
Graphene analogue and various topological phases in BaBiO_3_ bilayer (BBL) heterostructures. (**a**) Atomic configuration of the B-site atoms in the Ba*M*^I^O_3_/BBL/Ba*M*^II^O_3_ heterostructure. (**b**) Massless/massive Dirac cones and the corresponding topological phases in the BBL heterostructure as a function of spin-orbit coupling (SOC) (λ_SOC_) and the sublattice potential difference (Δ). The electronic band structure of the symmetric BaZrO_3_/BBL/BaZrO_3_ heterostructure (**c**) without and (**d**) with SOC, and (**e**) the topologically protected edge state of the quantum spin Hall (QSH) phase along the zigzag edge. The band structure of the asymmetric BaZrO_3_/BBL/BaHfO_3_ heterostructure (**f**) without and (**g**) with SOC, and (**h**) the Berry curvature that is alternating at each valley and indicating the quantum valley Hall (QVH) phase.

The maximum band-gap tunability of a Dirac cone, which is essential for topological electronics but barely achievable in graphene^12^, can be realized in a (111)-oriented BaBiO_3_ bilayer (BBL) oxide heterostructure. The confluence of oxide heterostructures^13–15^, Dirac materials^16^, and topological quantum matters^17–19^ produces a graphene analogue with a tunable band-gap, which links directly to the tunable topological phase. As shown in Fig. 1(a), in the crystal structure of the BBL, the Bi atom forms a buckled honeycomb network. Considering the Bi^4+^ (6*s*^1^) configuration of BaBiO_3_, the half-filled 6*s*-orbital in the honeycomb lattice inherently produces a Dirac cone at points *K* and *K*′ in the hexagonal Brillouin zone. The large SOC stems from the strong hybridization between the neighboring Bi 6*s*- and 6*p*-orbitals. Here we note that the bulk BaBiO_3_ was reported to be a three-dimensional topological insulator due to the large SOC of the Bi 6*p*-orbitals^19^. Importantly, the abundant order parameters of the sandwiching oxide perovskite materials can break various symmetries of BBL, allowing it to possess various topological phases.

## Results

### Various topology in oxide heterostructures

Specifically, Ba*M*^I^O_3_/BBL/Ba*M*^II^O_3_ heterostructures stacked along the [111]-direction are considered (Fig. 1(a)). The simple design principle applied for construction of the target system is that BBL provides a graphene-like low-energy electronic degree of freedom, and the sandwiching perovskite oxides, Ba*M*^I^O_3_ and Ba*M*^II^O_3_, determine the symmetry of the BBL. Therefore, the topological phase and band-gap of the Dirac cones rely completely on the choice of the *M*^I^ and *M*^II^ atoms. Given the wide variety of choice as *M*^I^ and *M*^II^ atoms, rich physics, including non-trivial band topology, is accessible in this oxide heterostructure platform.

When the *M*^I^ and *M*^II^ atoms are of the same species, and their perovskite form, Ba*M*^I,II^O_3_, has a para-electric, non-magnetic insulating phase, the BBL has the full space-group symmetry of the buckled honeycomb lattice and time-reversal symmetry. Subsequently, a gapless Dirac cone, the fundamental building block in this work, appears. After containing the SOC, the Dirac cone acquires a mass-gap and the QSH phase emerges, which exactly realizes the Kane-Mele model^9^. Alternatively, if we choose different *M*^I^ and *M*^II^ atoms while preserving the time-reversal symmetry, we can selectively break the inversion symmetry of the BBL heterostructure. The break in the inversion symmetry of the BBL resulting from using two different neighboring oxides is equivalent to the sublattice symmetry breaking of the buckled honeycomb lattice. As a result, the Dirac cone acquires a sublattice-dependent mass-gap, and the system adopts the QVH phase^10^. With no inversion symmetry, SOC can exhibit extra valley-dependent spin splitting at the gapped Dirac cone and introduce spin-valley coupling, which is frequently seen in the monolayer transition metal dichalcogenide series^20^. These features are summarized in Fig. 1(b), and the corresponding *ab-initio* calculations are shown in Fig. 1(c–h).

### Electronic structures

For the symmetric BBL configuration where *M*^I^ = *M*^II^ = Zr, the electronic structures without and with SOC are calculated in Fig. 1(c),(d), respectively. Gapless linear band dispersion appears inside the large band-gap of the sandwiching oxide, BaZrO_3_, and the low-energy band structure is governed by the Bi 6*s*-orbital Dirac cone (Fig. 1(c)). When SOC is included, a band-gap of 79 meV opens at the Dirac point (Fig. 1(d)); opening of a band-gap by SOC in an otherwise gapless Dirac cone indicates the presence of a Kane-Mele type topological insulator. The unexpectedly large SOC-induced gap in the *s*-orbital system is attributed to the strong Bi 6*s*-6*p* hybridization and the large atomic SOC of the Bi 6*p*-orbital (for details on the tight-binding analysis and SOC, see Supplementary Information). The topologically protected edge state, shown in Fig. 1(e), of the ribbon geometry with the zigzag edge confirms the QSH phase of the symmetric BBL heterostructure via the bulk-boundary correspondence. The same QSH phase of the symmetric configuration is attainable with other sandwiching perovskite compounds, including a Sr-based heterostructure in which all of the Ba atoms are replaced by Sr atoms. These are listed in Table 1, and the band-gap ranges from 45 to 100 meV. Therefore, the QSH phase in the symmetric BBL configuration is detectable and usable at room temperature.

**Table 1:** Lattice parameters and QSH gap in symmetric heterostructures.

Sandwiching layer	lattice parameter (Å)	QSH gap at *K* (meV)	Sandwiching layer	lattice parameter (Å)	QSH gap at *K* (meV)
BaTiO_3_	4.034	108	SrTiO_3_	3.943	102
BaZrO_3_	4.255	79	SrZrO_3_	4.196	89
BaSnO_3_*	4.187	72	SrSnO_3_*	4.111	81
BaHfO_3_	4.202	49	SrHfO_3_	4.140	59
BaCeO_3_	4.473	60	SrCeO_3_	4.427	45

The Ba- and Sr-based heterostructures are listed. All lattice parameters are calculated with the ideal perovskite structure. Lattice parameters of BaBiO_3_ and SrBiO_3_ are 4.421 Å and 4.366 Å, respectively.

*Because of the band-gap underestimation of BaSnO_3_ and SrSnO_3_ in DFT, the QSH gap appearing at points K and K′ is located above the Fermi level, when the Bi honeycomb lattice is sandwiched by BaSnO_3_ or SrSnO_3_ layers.

Figure 1(f),(g) depict the electronic structures without and with SOC, respectively, for the asymmetric BBL configuration where *M*^I^ = Zr and *M*^II^ = Hf. The structural inversion asymmetry induced by sandwiching with different oxides produces the potential difference at each sublattice site of the buckled honeycomb structure, and results in a QVH phase with a direct band-gap of 0.92 eV at the Dirac point (Fig. 1(f)). In the asymmetric BBL configuration, the band topology is characterized by the valley-Chern number, and the QVH phase is verified by calculating the valley-contrasting Berry curvature^10^ as shown in Fig. 1(h). The Berry curvature at each *K* and *K*′ valley has the opposite sign, leading to the non-zero valley-Chern number. As SOC is turned on, extra valley-dependent spin splitting at both the conduction and valence band edges occurs by 103 and 29 meV, respectively (Fig. 1(g)). In addition to the usefulness of the QVH phase for topological electronics, this interesting spin-valley coupling might be available for spin-valleytronics applications^20,21^; near *K* and *K*′, both valley-dependent Zeeman-type and valley-independent Rashba-type spin splitting occur on the out-of-plane and in-plane spin components, respectively (see Supplementary Information Fig. S2).

### Ferroelectric control of topological phases

Having revealed that two distinctive topological phases, QSH and QVH, take place in the same framework, the BBL heterostructure still has room for improvement in terms of topology-tunability; the QSH phase still cannot be converted into the QVH phase and vice versa within one system. Recalling that the topological phase of the BBL heterostructure is governed by its symmetry, topology-control is synonymous with symmetry-control in this system. Therefore, introducing a symmetry-breaking order parameter in the neighboring perovskite oxide makes it possible to control the topology of the BBL heterostructure. Because the corresponding symmetry that distinguishes the QSH and QVH phases is the spatial inversion, BBL sandwiched by ferroelectric perovskite oxides provides a way to control the inversion symmetry, and ultimately switch the QSH and QVH phases.

**Figure 2:**
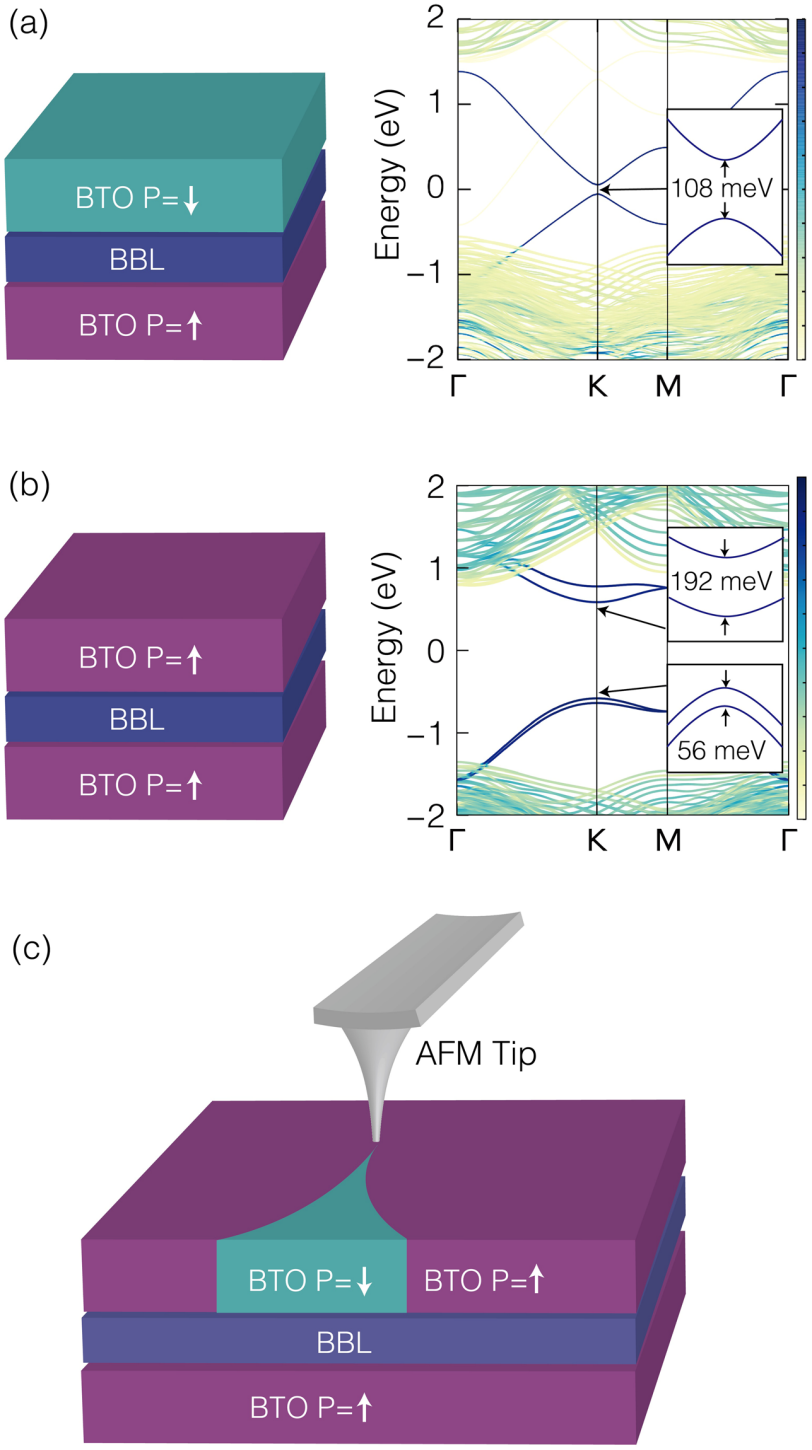
Ferroelectric control between QSH and QVH phases. Ferroelectric switching alters the topological phase of the BaTiO_3_/BBL/BaTiO_3_ heterostructure: (**a**) anti-parallel and (**b**) parallel polarization correspond to the QSH and QVH phases, respectively. (**c**) The ferroelectric domain boundary controlled using the atomic force microscope (AFM) tip generates the QSH/QVH topological domain boundary.

By sandwiching the well-known ferroelectric BaTiO_3_ with the [111] polarization direction, we can construct a BaTiO_3_/BBL/BaTiO_3_ heterostructure as a unified system hosting and controlling both the QSH and QVH phases. Depending on the relative polarization direction of the BaTiO_3_ on either side of the BBL, the inversion symmetry can be either conserved or broken; if the polarization direction is the opposite (same), the inversion symmetry of the BBL heterostructure is conserved (broken). As a result, the QSH (QVH) phase with a band-gap of 108 meV (1.17 eV) arises within the anti-parallel (parallel) polarization configuration, as shown in Fig. 2(a),(b)). Moreover, by developing a ferroelectric domain boundary, a topological domain boundary is induced, in which a topologically protected edge state emerges and the topological charge flows. Suppose that we prepare a BaTiO_3_/BBL/BaTiO_3_ heterostructure that initially has a parallel polarization configuration. As the polarization of the upper BaTiO_3_ layer starts to be flipped using an atomic-force microscope (AFM) tip and a ferroelectric domain boundary is generated, the topological domain boundary between the QSH and QVH phases develops simultaneously (see Fig. 2(c)). This controllability^22^ enables us to manipulate the conducting path of the topological charge in a non-volatile, reconfigurable way, which is an important operation in topological electronics.

## Discussion

**Figure 3:**
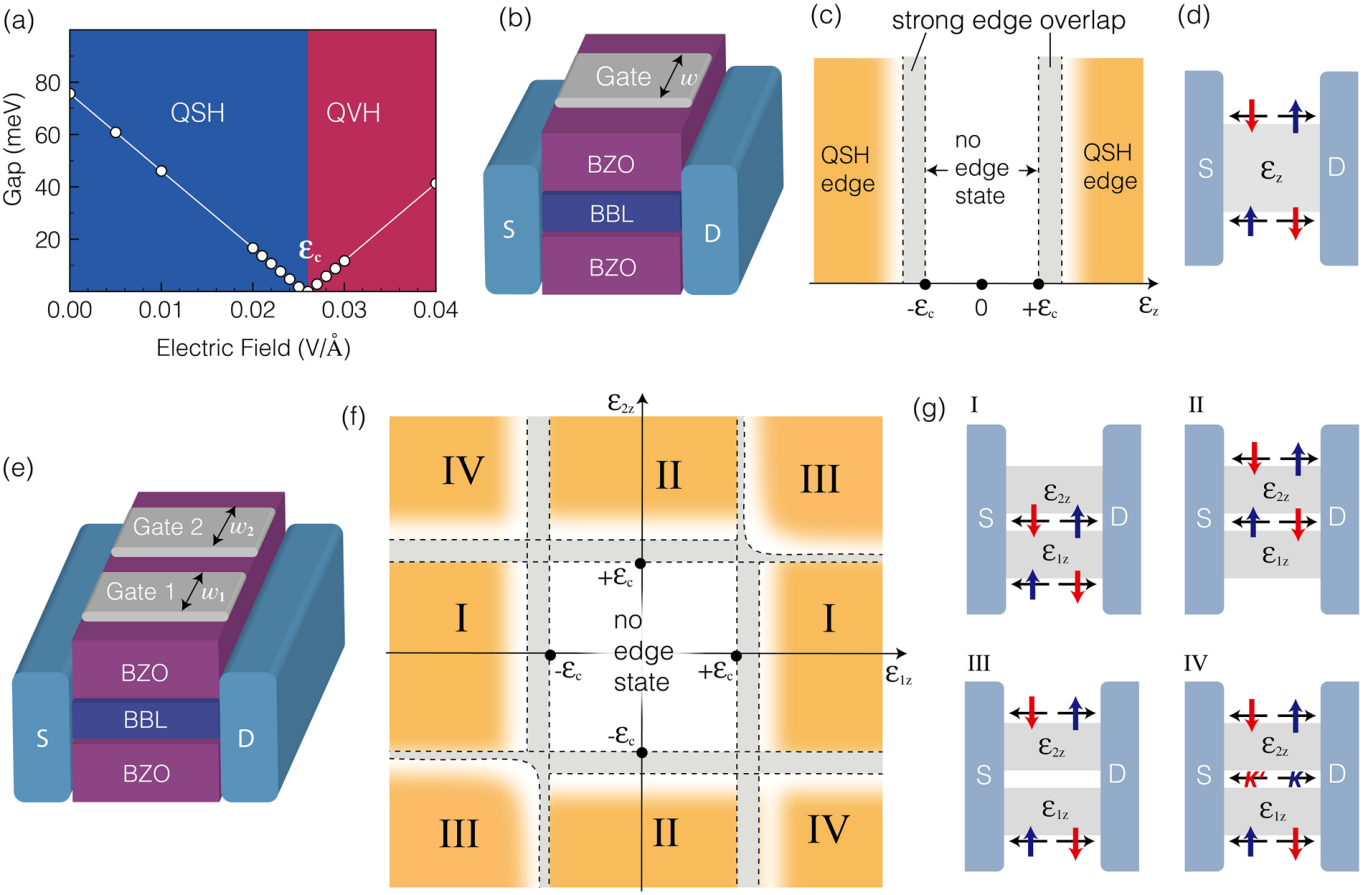
Stability of QSH phase and design of topological electronic field effect transistors. (**a**) The stability of the QSH phase in the symmetric BBL heterostructure is tested by applying a vertical electric field. (**b**) The single-gated transistor and (**c**) edge states as a function of the gate electric field are depicted schematically. (**d**) For a large gate electric field, the spin-momentum locked QSH edge states appear at both sides of the QSH/QVH boundary. (**e**) The double-gated transistor is sketched, and (**f**) the various topological domain boundaries are tuned by the gate electric fields. (**g**) The corresponding topological edge configurations are shown. The grey area in (**c**) and (**f**) indicates the strong edge overlapped region.

A major premise in this work is the Bi^4+^ charge state in the buckled honeycomb lattice; the fundamental building block—the 6*s*-orbital Dirac cone—and the subsequent topological phases hinge entirely on it. However, Bi^4+^ is unstable in bulk BaBiO_3_, in which the charge ordering of Bi^3+^ and Bi^5+^ is accompanied by breathing instability of the BiO_6_ octahedron, and the Bi^3+^ and Bi^5+^ planes alternate along the [111]-direction^23^. If the Bi^4+^ charge state is unstable in the BBL heterostructure, the QSH phase seen in the symmetric configuration becomes unstable and transforms into the QVH phase by spontaneously breaking the sublattice symmetry. Therefore, it is important to verify the robustness of Bi^4+^ and the graphene-like electronic structure in the BBL heterostructure against the charge ordering instability. We applied a small external electric field along the [111]-direction of the BaZrO_3_/BBL/BaZrO_3_ slab geometry to examine whether a small perturbation can trigger the charge ordering and induce the QVH phase abruptly. As shown in Fig. 3(a), the QSH band-gap of 75.7 meV decreases continuously, closes at *ε*_c_ = 26 mV/Å, and re-opens as the field strength increases. At the gap-closing point, band inversion occurs and the topology of the system changes from the QSH to the QVH phase. Consequently, unlike the bulk BaBiO_3_, the Bi^4+^ charge state and QSH phase in the symmetric configuration of the BBL heterostructure remain intact under a small external perturbation. Recent experiments examining BaBiO_3_ thin films showed that the charge ordering instability is suppressed as the film thickness decreases^24^, which is consistent with our result.

This stability test revealed one more appealing aspect; the Bi^4+^ charge state is not only sufficiently stable to sustain multiple Dirac cones, but is also susceptible to easy manipulation of the band-gap and topological phase by a small electric field. The QSH gap is closed by an external field of *ε*_c_ = 26 mV/Å and the band topology is switched to the QVH phase at greater field strengths. The large buckling angle in the BBL honeycomb lattice and the latent Bi^3+/5+^-tendency seem to be responsible for the easy tunability, and make the system a potential platform for topological electronics via the electric field. Taking advantage of the easy controllability of the band-gap and topology of the BBL heterostructure, we designed a topological electronic field-effect-transistor^25^, as shown in Fig. 3(b–g). The single-gated transistor sketched in Fig. 3(b) can develop the QSH/QVH domain boundary, possibly leading to the QSH edge state when the gate electric field *ε*_z_ > *ε*_c_. Considering the finite width *W* of the gate electrode, the edge states located at both sides of the topological domain boundary can overlap if the band-gap of the QVH phase induced by *ε*_z_ is small. Given that the penetration length of the edge state into the insulating bulk is $\frac{{v}_{F}}{{E}_{g}}$, where *v*_*F*_ is the Fermi velocity of the Dirac cone (3.55 × 10^5^ m/s in the BBL heterostructure) and *E*_g_ is the band-gap, strong edge overlap occurs when $\frac{W}{2}\lesssim \frac{{v}_{F}}{{E}_{g}}$. For example, if *W* = 50 nm and *ε*_c_ < *ε*_z_ ≤ 29.2 mV/Å, the QSH edge states on both sides easily interact via the shallow-gapped bulk region, hampering its ability to carry a topological charge through the topological domain boundary. Therefore, a gate electric field well above *ε*_c_ can host the spin-momentum-locked QSH edge states (see Fig. 3(c),(d)).

The double-gated system sketched in Fig. 3(e) can give rise to various topological domain boundary configurations and their corresponding topological edge states. As well as the QSH/QVH boundary, the topological domain boundary between two different QVH phases, whose non-zero valley-Chern numbers have the opposite sign, also appears depending on the gate electric fields *ε*_1z_ and *ε*_2z_ (Fig. 3(f)). Again, due to the finite width of the two gate electrodes *W*_1_ and *W*_2_, a strong edge-overlapped region exists, and is shown in grey in Fig. 3(f). With |*ε*_1z_| < *ε*_c_ < |*ε*_2z_| (|*ε*_2z_| < *ε*_c_ < |*ε*_1z_|), the double-gated transistor behaves in the same way as the single-gate transistor, and the strong edge-overlapped region is determined by $\frac{{W}_{1}}{2}\lesssim \frac{{v}_{F}}{{E}_{1g}}$ $(\frac{{W}_{2}}{2}\lesssim \frac{{v}_{F}}{{E}_{2g}})$. When both gate electric fields exceed *ε*_c_ and are aligned in parallel, the QVH phase is formed in a region whose effective width is *W*_1_ + *W*_2_; thus, the condition for the strong edge overlap between the QSH edge states becomes $\frac{{W}_{1}+{W}_{2}}{2}\lesssim \frac{{v}_{F}}{{E}_{1g}}+\frac{{v}_{F}}{{E}_{2g}}$. Interestingly, two gate electric fields exceeding *ε*_c_ but with an anti-parallel alignment develop two QVH phases possessing the opposite valley-Chern numbers. As a result, the valley-momentum-locked QVH edge states appear along the inner QVH domain boundary^26,27^, together with the QSH edge states along the outer QSH/QVH boundary. The existence and position of various non-trivial edge states can be controlled by the gate electric fields in the double-gated transistor (Fig. 3(g)). In an array of multiple topological electronic transistors based on the BBL heterostructure, we can tailor the conducting path that carries two different topological charges^28^.

In conclusion, we show that the hetrostructure of a (111)-oriented BaBiO_3_ bilayer sandwiched between perovskite oxides can host QSH and QVH phases with the choice of sandwiching materials, and is therefore a promising platform of topological electronics. Although the overall discussion focuses on controlling the inversion symmetry, a BBL heterostructure sandwiched by various ferroic perovskite oxides will likely serve as a general scheme for hosting many controllable topological phases. For example, by introducing a ferromagnetic sandwiching oxide, we can break the time-reversal symmetry of the BBL in two different ways. With parallel ferromagnetic moments on both sides, the BBL honeycomb lattice is subject to a constant Zeeman field, possibly generating the quantum anomalous Hall phase by breaking the time-reversal symmetry^4^. For the anti-parallel ferromagnetic moments, a sublattice-dependent staggered Zeeman field arises, leading to the so-called quantum spin-valley Hall phase by breaking both the time-reversal and inversion symmetries but conserving the composite of the two symmetries^29^. For the BBL sandwiched by multi-ferroic perovskite oxides, we can control both the spatial inversion and time-reversal symmetries simultaneously^30^. The potential to make a three-dimensional integrated superlattice composed of various topological compartments makes the BBL heterostructure even more fascinating.

## Methods

### First-principles calculations

The first-principles calculations were performed using the density functional theory with projector augmented wave potentials and the Perdew-Burke-Ernzerhof exchange correlation functional as implemented in VASP code^31,32^. The plane-wave energy cutoff was set at 400 eV, and 12 × 12 × 1 mesh was used for momentum space sampling. The atomic structures were relaxed with the force criteria of 0.005 eV/Å. The wannier90 code was used to construct the maximally-localized Wannier function^33^, in which four Bi *s*-projectors (including spin components) were used to reproduce the band structures near the Fermi level. The Berry curvature was calculated with the linear response Kubo formula, and the tight-binding Hamiltonian of strip geometry was constructed using the obtained Wannier functions and hopping parameters.

### Data Availability

The datasets generated during and/or analysed during the current study are available from the corresponding author on reasonable request.

## Electronic supplementary material

Supplementary Information
