# Supplementary material for: Graphene analogue in (111)-oriented BaBiO3 bilayer heterostructures for topological electronics
Source: Sci Rep. 2018 Jan 11;8:555. doi: 10.1038/s41598-017-19090-3 (PMC5765078; doi:10.1038/s41598-017-19090-3)
Supplement: Supplementary file 1 — Supplementary Information [file 41598_2017_19090_MOESM1_ESM.pdf]

Supplementary Informations for

Graphene analogue in (111)-oriented $BaBiO_3$ bilayer heterostructures for topological electronics

Rokyeon Kim[1,2], Jaejun Yu[2], and Hosub Jin[3,*]

[1]Center for Correlated Electron Systems, Institute for Basic Science (IBS), Seoul 08826, Korea

[2]Department Physics and Astronomy, Seoul National University, Seoul 08826, Korea

[3]Department of Physics, Ulsan National Institute of Science and Technology (UNIST), Ulsan 44919, Korea

Spin texture of the asymmetric BBL heterostructure

Figure S1 shows the spin texture of the asymmetric BBL heterostructure, $BaZrO_3$/BBL/$BaHfO_3$. The spin split-off valence bands and conduction bands are denoted by VB-1, VB, CB, and CB+1. At points *K* and *K′*, the spins are fully aligned along the out-of-plane direction due to the three-fold rotational symmetry. Near these points, Rashba-type in-plane spin alignment develops. The spin textures are reversed completely for the (VB-1, VB) and (CB, CB+1) pairs.

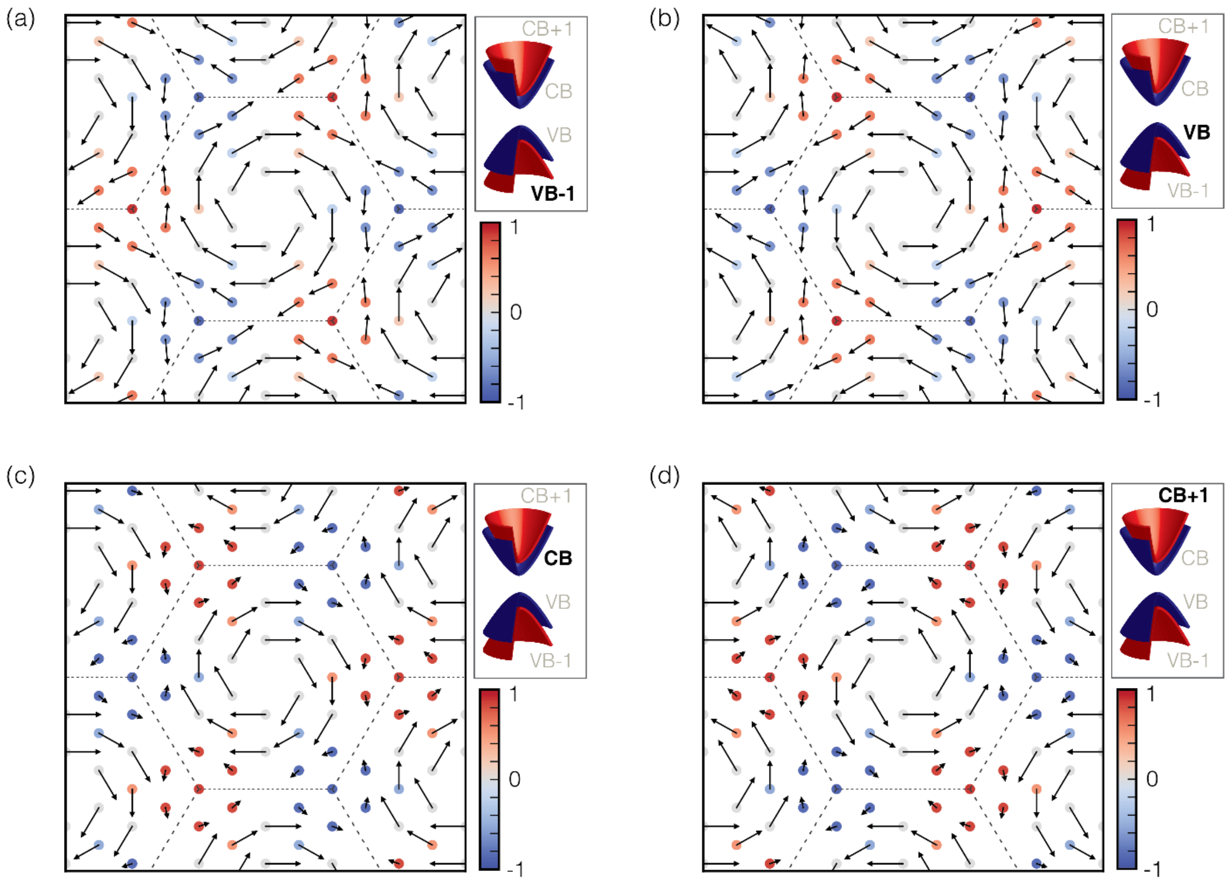

Figure S1| Spin texture in the BaZrO3/BBL/BaHfO3 heterostructure of (a) VB-1, (b) VB, (c) CB, and (d) CB+1.

Tight-binding analysis and SOC

Assuming the ideal BiO_6 octahedron, the SOC-induced hopping process within the next-nearest-neighbor (NNN) 6*s*-orbital is illustrated in Fig. S2, where the *xyz*-axis denotes the local coordinate of the octahedron. Due to the lack of orbital degrees of freedom in the Bi 6*s*-orbital, there is no on-site SOC term. Consequently, the lowest-order SOC is induced by the strong hybridization between the 6*s*- and 6*p*-orbitals and the large on-site SOC of the Bi 6*p*-orbital,

denoted by $V_{sp\sigma}$ and λ, respectively. As shown in Fig. S2, the s-orbital with spin-β at site-A1 hops to the p_x-orbital with the same spin at the nearest-neighbor site-B, for which the amplitude is proportional to $V_{sp\sigma}$. Then, the p_x-orbital is transformed into the p_y-orbital with spin-α by the on-site SOC at the same site. Finally, the p_y-orbital hops back to the s-orbital at the NNN site-A2, conserving the same spin-α:

$$|6s\otimes\beta\rangle\mathrm{A1}\xrightarrow{-V_{sp\sigma}}|6p_x\otimes\beta\rangle\mathrm{B}\xrightarrow{\frac{i\lambda}{2}\sigma_z^{\alpha\beta}}|6p_y\otimes\alpha\rangle\mathrm{B}\xrightarrow{-V_{sp\sigma}}|6s\otimes\alpha\rangle\mathrm{A2}$$

In short, the NNN SOC hopping process from site-A1 to A2 is $i\frac{\lambda_{\mathrm{SOC}}}{3}C_{A2,\alpha}^{\dagger}\sigma_z^{\alpha\beta}C_{A1,\beta}$, where $\lambda_{\mathrm{SOC}}=\frac{3V_{sp\sigma}^2}{2(\epsilon_p-\epsilon_s)^2}\lambda$. The complete expression for the SOC Hamiltonian under the global coordinate system, where the z-axis is parallel to the [111] direction, is written as follows:

$$\mathcal{H}_{\mathrm{SOC}}=\frac{i}{3\sqrt{3}}\lambda_{\mathrm{SOC}}\sum_{\langle\langle i,j\rangle\rangle\alpha\beta}\nu_{ij}C_{i\alpha}^{\dagger}\sigma_z^{\alpha\beta}C_{j\beta}-\frac{2i}{3\sqrt{6}}\lambda_{\mathrm{SOC}}\sum_{\langle\langle i,j\rangle\rangle\alpha\beta}\mu_{ij}C_{i\alpha}^{\dagger}(\vec{\sigma}\times\vec{d}_{ij})_z^{\alpha\beta}C_{j\beta}\ ,$$

where $\nu_{ij}=\begin{cases}+1 \text{ if electron makes a right turn to the NNN site}\\-1 \text{ if electron makes a left turn to the NNN site}\end{cases}$,

$\mu_{ij}=\begin{cases}+1 \text{ for hopping between } A \text{ sublattice}\\-1 \text{ for hopping between } B \text{ sublattice}\end{cases}$, and $\vec{d}_{ij}$ is the unit vector connecting site-j to site-i.

This process looks similar to that observed in silicene, where the Si pz-orbital forms the Dirac cone and SOC is induced by hybridization of the p_z and $p_{x/y}$ orbitals in the buckled structure[1]. Compared with silicene, however, hopping from Bi 6s to Bi 6p is always possible regardless of the buckling angle in the BBL.

By combining nearest-neighbor hopping between the Bi 6*s*-orbital and NNN SOC hopping, we can obtain the minimal tight-binding Hamiltonian to describe the BBL heterostructures. Expanding around points *K* and *K*′, the low-energy effective Hamiltonian is written as

$$\mathcal{H}_{\text{eff}}^{\eta} = v_F(\eta k_x\tau_x - k_y\tau_y)\otimes\sigma_0 - \lambda_{\text{SOC}}\eta\tau_z\otimes\sigma_z - \lambda_{\text{R}}\tau_z\otimes(k_x\sigma_y - k_y\sigma_x),$$

where $v_F = -\frac{\sqrt{3}}{2}V_{ss\sigma}$, $\lambda_{\text{R}} = \frac{\sqrt{6}V_{sp\sigma}^2}{4(\epsilon_p-\epsilon_s)^2}\lambda$, $\eta = \pm 1$ for valleys *K* and *K*′, and σ_i and τ_i are the Pauli matrices for the real spin and sublattice pseudo-spin degrees of freedom, respectively.

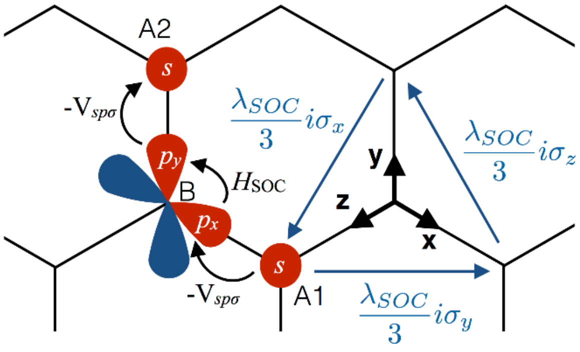

Figure S2| SOC-induced hopping process within the next-nearest-neighbor 6*s*-orbitals.

REFERENCES

1 Liu, C.-C., Jiang, H. & Yao, Y. Low-energy effective Hamiltonian involving spin-orbit coupling in silicene and two-dimensional germanium and tin. *Phys. Rev. B* **84**, 195430, doi:10.1103/PhysRevB.84.195430 (2011).
